# Differential responses of ammonia-oxidizing archaea and bacteria to long-term fertilization in a New England salt marsh

**DOI:** 10.3389/fmicb.2012.00445

**Published:** 2013-01-22

**Authors:** Xuefeng Peng, Erik Yando, Erica Hildebrand, Courtney Dwyer, Anne Kearney, Alex Waciega, Ivan Valiela, Anne E. Bernhard

**Affiliations:** ^1^Department of Biology, Connecticut CollegeNew London, CT, USA; ^2^The Ecosystems Center, Marine Biological LaboratoryWoods Hole, MA, USA

**Keywords:** *amo*A, TRFLP, Great Sippewissett Marsh, fertilization, salt marsh

## Abstract

Since the discovery of ammonia-oxidizing archaea (AOA), new questions have arisen about population and community dynamics and potential interactions between AOA and ammonia-oxidizing bacteria (AOB). We investigated the effects of long-term fertilization on AOA and AOB in the Great Sippewissett Marsh, Falmouth, MA, USA to address some of these questions. Sediment samples were collected from low and high marsh habitats in July 2009 from replicate plots that received low (LF), high (HF), and extra high (XF) levels of a mixed NPK fertilizer biweekly during the growing season since 1974. Additional untreated plots were included as controls (C). Terminal restriction fragment length polymorphism analysis of the *amo*A genes revealed distinct shifts in AOB communities related to fertilization treatment, but the response patterns of AOA were less consistent. Four AOB operational taxonomic units (OTUs) predictably and significantly responded to fertilization, but only one AOA OTU showed a significant pattern. Betaproteobacterial *amo*A gene sequences within the *Nitrosospira*-like cluster dominated at C and LF sites, while sequences related to *Nitrosomonas* spp. dominated at HF and XF sites. We identified some clusters of AOA sequences recovered primarily from high fertilization regimes, but other clusters consisted of sequences recovered from all fertilization treatments, suggesting greater physiological diversity. Surprisingly, fertilization appeared to have little impact on abundance of AOA or AOB. In summary, our data reveal striking patterns for AOA and AOB in response to long-term fertilization, and also suggest a missing link between community composition and abundance and nitrogen processing in the marsh.

## INTRODUCTION

Oxidation of ammonia to nitrite is a critical process in nitrogen cycling and is carried out by a suite of distinct microorganisms within the bacterial and archaeal domains. The fate of nitrogen is of particular importance in nitrogen-sensitive coastal systems, such as estuaries and salt marshes, where primary productivity is typically nitrogen-limited (Valiela and Teal, 1974; Howarth, 1988). Nitrogen-cycling processes in salt marshes, therefore, play a significant role in the health and preservation of coastal habitats, yet our understanding of the ecology of the organisms responsible and their responses to perturbations is still evolving.

Over the last decade, researchers have made great progress in describing the diversity and distribution of ammonia-oxidizing bacteria (AOB) in estuaries (see review by Bernhard and Bollmann, 2010) and to a lesser extent in salt marshes (Dollhopf et al., 2005; Moin et al., 2009). Surveys of 16S rRNA genes and ammonia monooxygenase genes (coding for the enzyme responsible for the first step in ammonia oxidation) in estuaries and salt marshes indicate highly similar AOB communities in geographically distinct habitats that are typically dominated by sequences most closely related to *Nitrosospira* spp., although representative AOB have yet to be obtained in pure culture. Most studies have also reported salinity to be a major factor regulating the distribution and diversity of AOB in these coastal systems (Francis et al., 2003; Bernhard et al., 2005; Ward et al., 2007). However, with the recent discovery of ammonia-oxidizing archaea (AOA; Könneke et al., 2005; Treusch et al., 2005) and their apparent wide distribution in many habitats, the relative importance of AOB to nitrification and their potential interactions with AOA have been brought into question.

The diversity and distribution of AOA have begun to be explored in estuaries and salt marshes, but, unlike the AOB, no consistent patterns or common regulatory factors have been identified. Although archaeal *amo*A gene sequences from both the water column/sediment and soil/sediment clusters as described in Francis et al. (2005) have been recovered from estuaries and salt marshes, to date, most archaeal *amo*A genes fall into the water column/sediment cluster, but the diversity of sequence types is quite high (Bernhard and Bollmann, 2010). Furthermore, factors that have been identified as potentially regulating their distribution are many, including salinity, temperature, pH, dissolved oxygen, nitrogen, and net primary productivity (Beman and Francis, 2006; Sahan and Muyzer, 2008; Santoro et al., 2008; Moin et al., 2009). Thus, our understanding of AOA communities and what regulates their diversity and distribution, and ultimately, their activity in these important coastal ecosystems is still rudimentary.

Manipulating the environment and following changes in communities can be a powerful approach to help tease apart important regulatory factors. Using this approach, Lage et al. (2010) identified changes in AOB communities in relation to short-term N and P additions in a salt marsh. Others have taken a similar approach in soil systems to study changes in AOB communities (Chu et al., 2007, 2008; Shen et al., 2011), and have generally reported significant shifts in activity, community composition, and/or abundance. However, studies of AOA in response to fertilization have yielded conflicting results (Kelly et al., 2011; Verhamme et al., 2011; Wu et al., 2011).

In this study, we explored the diversity and distribution of AOA and AOB in a series of salt marsh plots that have received different levels of commercial fertilizer for nearly 40 years. The plots were originally fertilized to study the capacity of salt marshes to remove and retain nutrients (Valiela et al., 1973). Significant effects of fertilization on other components of the marsh, including vegetation (Valiela et al., 1975, 1976), invertebrates (Meany et al., 1976; Sarda et al., 1996), diatoms (Van Raalte et al., 1976), and nitrogen-cycling processes (Van Raalte et al., 1974; Hamersley and Howes, 2005) have been documented. More recently, Bowen et al. (2011) reported little evidence of fertilization impacts on bacterial and denitrifying communities in the marsh. To further the work of Bowen et al. (2011) on describing effects of fertilization on microbial communities, we report the first investigation of fertilization impacts on nitrifying microorganisms in the marsh. Two of the questions addressed by our study are: (1) Are there major shifts in AOA and AOB communities that correspond with fertilization treatments? and (2) Do specific populations of AOA or AOB respond predictably to fertilization?

Known effects of experimental enrichment on the nitrogen cycle in salt marsh sediments lead to a prediction that there must be significant responses by ammonia oxidizers to the supply of nitrogen entering salt marsh ecosystems. We know that denitrification rates increase as N supply increases (Hamersley and Howes, 2005), and concentrations of ammonium are consistently higher in fertilized plots relative to those in control plots (Hamersley and Howes, 2005). Since denitrification in the fertilized salt marsh sediments is supported largely by *in situ* oxidation of ammonium (Hamersley and Howes, 2005), we anticipated an increase in abundance of ammonia oxidizers accompanied by shifts in community composition.

## MATERIALS AND METHODS

### SITE DESCRIPTION

**FIGURE 1:**
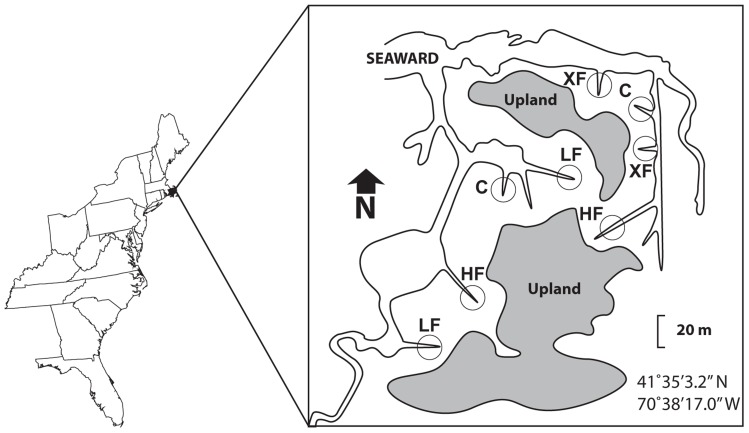
**Map of study plots in the Great Sippewissett Marsh, Falmouth, MA, USA**. C, control plots with no fertilizer added; LF, low fertilization; HF, high fertilization; XF, extra high fertilization (levels of fertilization can be found in Section “Materials and Methods”).

This study was conducted in the Great Sippewissett Marsh, Falmouth, MA, USA (**Figure 1**). Circular plots (10 m diameter) of salt marsh vegetation have been fertilized every 2 weeks during the growing season since the early 1970s (Valiela et al., 1973, 1975). Three different levels of mixed NPK fertilizer were applied to each set of two replicate plots: low (LF; 8.4 g/m^2^/week), high (HF; 25 g/m^2^/week), and extra high (XF; 75 g/m^2^/week). The fertilizer is a commercially available sewage sludge fertilizer (10% N, 6% P, 4% K by weight). Forty percent of the N is inorganic, but only 28% is immediately soluble (Valiela et al., 1973). An additional two replicate plots were left unfertilized as controls (C). The rates of fertilization correspond to nitrogen loading rates of 12 (C), 182 (LF), 532 (HF), and 1572 (XF) kg N/ha/year (Fox et al., 2012), and include atmospheric deposition on the marsh surface. The plots are located within an experimental area of 10 ha. Areas within the plots receiving frequent (daily) tidal inundation and dominated by *Spartina alterniflora* are designated as low marsh habitat. Areas above mean high tide and dominated by *S. patens* (in control plots) or *Distichlis spicata* (in XF plots) and are designated as high marsh habitat. More detailed information regarding recent vegetation cover within the plots is reported elsewhere (Fox et al., 2012).

### SAMPLE COLLECTION

Duplicate sediment cores (6.5 cm in diameter) were collected from low marsh habitat in each of eight experimental plots described above in July 2009 during low tide. Within the C and XF plots, two additional cores were collected from the high marsh habitat. Cores were kept on ice in the dark for several hours during transport to the lab. Salinity was measured by a handheld refractometer from pore water extracted from each core by centrifugation.

### DNA EXTRACTION

Subsamples (ca. 0.5 g) were taken from sediment cores at a depth of 0–2 cm, and stored at -80°C. DNA was extracted using the PowerSoil DNA Isolation Kit (MO BIO, Carlsbad, CA, USA) according to the manufacturer’s protocol. DNA concentrations were measured spectrophotometrically using a SmartSpec spectrophotometer (Bio-Rad, Hercules, CA, USA) and fluorometrically using a Quant-It fluorometer (Invitrogen, Grand Island, NY, USA). Extracted DNA was also assessed by gel electrophoresis (1% agarose) with 0.1 μl/ml of ethidium bromide. All DNA extractions were performed on duplicate 0.5 g aliquots of sediment and the better of each extraction (assessed by quality and quantity) was selected for downstream analysis.

### TRFLP ANALYSIS

Archaeal *amo*A genes were amplified with Arch-AmoAF and Arch-AmoAR (Francis et al., 2005). Betaproteobacterial *amo*A genes were amplified with amoA-1F and amoA-2R-TC (Rotthauwe et al., 1997; Nicolaisen and Ramsing, 2002). Each 20 μl reaction contained 1× DreamTaq Buffer with KCl and (NH_4_)_2_SO_4_ (Fermentas, Inc., Glen Burnie, MD, USA), 320 ng/μl of bovine serum albumin, 3.75 mM of MgCl_2_, 0.2 mM of dNTPs, 0.5 U of DreamTaq DNA Polymerase (Fermentas), 0.4 μM of each primer, and approximately 2–10 ng DNA. Each forward primer was labeled at the 5′ end with 6-FAM (Eurofins MWG Operon, Huntsville, AL, USA) and all reactions were carried out in an iCycler (Bio-Rad). Archaeal *amo*A genes were amplified with the following cycle conditions: 30 s of initial denaturation at 95°C, followed by 35 cycles of 95°C for 15 s, 54°C for 20 s, and 72°C for 45 s, and ending with a final elongation at 72°C for 5 min. Betaproteobacterial *amo*A genes were amplified with the same parameters except the annealing temperature was 57°C.

Polymerase chain reaction (PCR) products were digested with 10 U of *Aci*I (NEB), 2 μl of 10× NEBuffer 3 in a 20-μl reaction volume for 6 h at 37°C. Following ethanol precipitation, 10 μl of formamide, 5 μl of filter-sterilized MilliQ water, and 0.2 μl of GeneScan 500 Size Standard (Applied Biosystems, Foster City, CA, USA) were added to the samples. Samples were analyzed at the Cornell University Life Sciences Core Laboratories Center using an Applied Biosystems 3730xl DNA Analyzer. Community fingerprints were analyzed using GeneMarker 1.4 (SoftGenetics, State College, PA, USA). Only terminal restriction fragments (TRFs) that correspond to a sequence either in our sequences reported here or from GenBank were included in the final community analyses. Although including only TRFs represented by a known sequence likely underestimates the community diversity in the terminal restriction fragment length polymorphism (TRFLP) analysis, particularly for the less well-described archaeal *amo*A diversity, we believe it minimizes artifacts that may significantly skew the analysis.

### *amo*A GENE CLONING AND SEQUENCING

Archaeal and betaproteobacterial *amo*A genes were amplified and cloned as previously described (Moin et al., 2009). A total of 14 clone libraries were constructed. Betaproteobacterial and archaeal *amo*A libraries were constructed from plots 3 (C), 5 (LF), 9 (HF), 6 (XF) in the low marsh and plots 7 (C) and 6 (XF) in the high marsh. An additional archaeal *amo*A clone library was constructed from low marsh in plot 8 (XF) for comparison between plots and an additional betaproteobacterial *amo*A clone library was constructed from a duplicate core collected from low marsh in plot 3 (C). Each library consisted of 96 randomly selected clones. Approximately 48–96 clones from each library were sequenced using the T3 primer and aligned in ARB (Ludwig et al., 2004). *In silico* TRF sizes were determined for all sequences.

### REAL-TIME PCR ANALYSES

Betaproteobacterial *amo*A genes were quantified as described in Bernhard et al. (2007). Archaeal *amo*A genes were quantified as described in Moin et al. (2009). Bacterial 16S rRNA genes were quantified as described in Bernhard et al. (2012). Average amplification efficiencies from at least three separate runs were 101.3 ± 13.3% (betaproteobacterial *amo*A genes), 107.4 ± 5.2% (archaeal *amo*A genes), and 91.7 ± 4.5% (bacterial 16S rRNA genes). We calculated copy numbers of *amo*A genes per gram of sediment by correcting the number of copies detected in each PCR for the DNA extraction volume obtained from a known amount of sediment for each sample. We also normalized *amo*A gene copies to μg DNA (measured fluorometrically) and to copies of bacterial 16S rRNA genes. We detected no significant differences in amount of DNA among fertilization treatments (ANOVA, *P* = 0.2)

### STATISTICAL ANALYSIS

ANOVA, *t*-test, Pearson correlation, and multiple regression analyses were performed in InStat v3.0b (GraphPad, La Jolla, CA, USA). Data from duplicate cores in each plot were averaged prior to statistical analyses. Gene abundance data were log transformed prior to analyses to alleviate heteroscedasticity.

Non-metric multidimensional scaling (NMS) was used to ordinate arcsine transformed TRFLP data with Sørensen’s distance measure (Bray–Curtis), using a stability criterion of 0.00001. All ordinations used the Autopilot mode set to slow and thorough, and a suggested iterative optimization procedure (McCune and Mefford, 1999). Final stress, final instability, number of iterations, difference from Monte Carlo test, recommended dimensionality, and final ordination scores were used to evaluate the results. Ordinations were rotated to maximize the correlation of fertilization rates on axis 1.

### SEQUENCE ANALYSIS

Sequences were compared to published sequences in GenBank using the Basic Local Alignment Search Tool (blastn) to identify related sequences and aligned using the sequence editor and Fast Align in ARB. All alignments were checked manually and regions of ambiguous alignments were excluded from the analysis. Phylogenetic relationships were analyzed by the neighbor-joining algorithm with the Kimura two-parameter correction in ARB. Confidence in tree topology was assessed by 100 bootstrap replicates using PHYLIP v.3.69. Sequences were checked for chimeras by comparing phylogenetic placement in trees constructed with the 5′ and the 3′ ends of the sequence. Pairwise sequence comparisons were calculated in ARB and operational taxonomic units (OTUs) were defined as sequences sharing ≥95% nucleotide sequence identity using MOTHUR (Schloss et al., 2009). Coverage of each clone library was calculated with the equation: *C* = 1 - (*n*/*N*) where *n* = number of singleton sequences and *N* = total number of sequences analyzed. A total of 526 betaproteobacterial *amo*A sequences and 310 archaeal *amo*A sequences were included in the final analysis. Sequence data have been submitted to the GenBank database under accession numbers JX283750–JX284059 (archaeal *amo*A) and JX306111–JX306636 (betaproteobacterial *amo*A).

## RESULTS

Salinity ranged from 23.6 ± 4.4 in the LF plots to 26.8 ± 1.8 psu in the C plots in the low marsh habitat and was not significantly different among fertilization treatments. In the high marsh plots, however, salinity was significantly lower (*P* = 0.0004) at the XF plots (11.7 ± 2.7 psu) compared to salinity in the control plots (31.0 ± 0.3 psu). Other variables, such as ammonium and sulfide, vary considerably among plots and a summary of these differences can be found elsewhere (Hamersley and Howes, 2005; Bowen et al., 2011).

### SHIFTS IN AOB COMMUNITY COMPOSITION

Terminal restriction fragment length polymorphism analysis of betaproteobacterial *amo*A genes revealed a total of 12 TRFs and an average of 8.3 TRFs per sample. TRF 130 and 192 were the most abundant overall. Only one TRF (472) was not identified in our sequence dataset, but this TRF represents *Nitrosomonas eutropha*, so we included it in our analysis.

**FIGURE 2:**
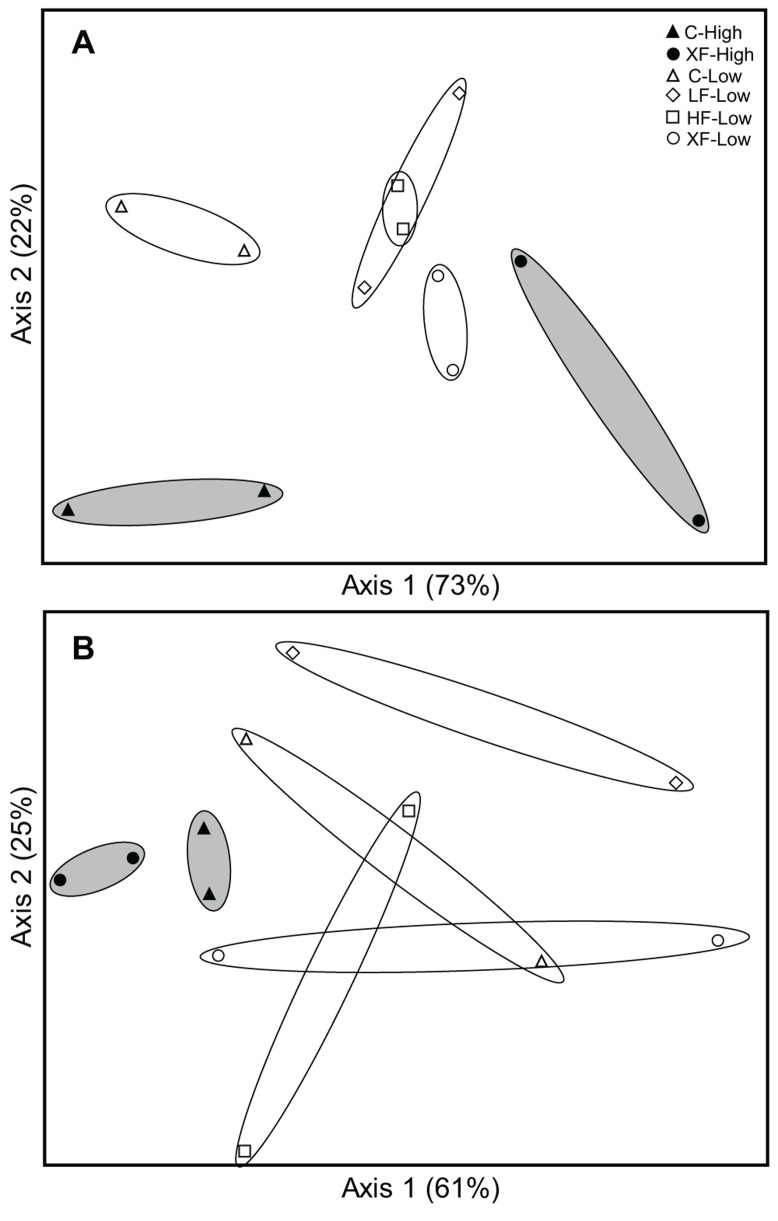
**Non-metric multidimensional scaling ordinations based on TRFLP profiles for betaproteobacterial *amo*A genes **(A)** and archaeal *amo*A genes **(B)****. Low marsh samples are represented by open symbols; high marsh samples are represented by filled symbols. Polygons (white-filled for low marsh, gray-filled for high marsh) are drawn around replicate samples in each plot. The percent variability explained by each axis is indicated parenthetically.

Analysis of betaproteobacterial *amo*A TRFLP patterns using NMS ordinations revealed distinct communities related to fertilization in both the low and high marsh habitats (**Figure 2A**). In the low marsh, there was generally a linear shift from AOB communities in control plots to AOB communities in XF plots, with AOB communities from LF and HF plots falling in between those from C and XF plots.

Correlation of ordination axis scores with environmental variables indicated that axis 1 was most strongly correlated with fertilization rate (*r* = 0.76, *P* < 0.004) and N loading rate (*r* = 0.76, *P* = 0.004), followed by salinity (*r* = -0.61, *P* = 0.03). Axis 2 was not significantly correlated with any of the variables.

**FIGURE 3:**
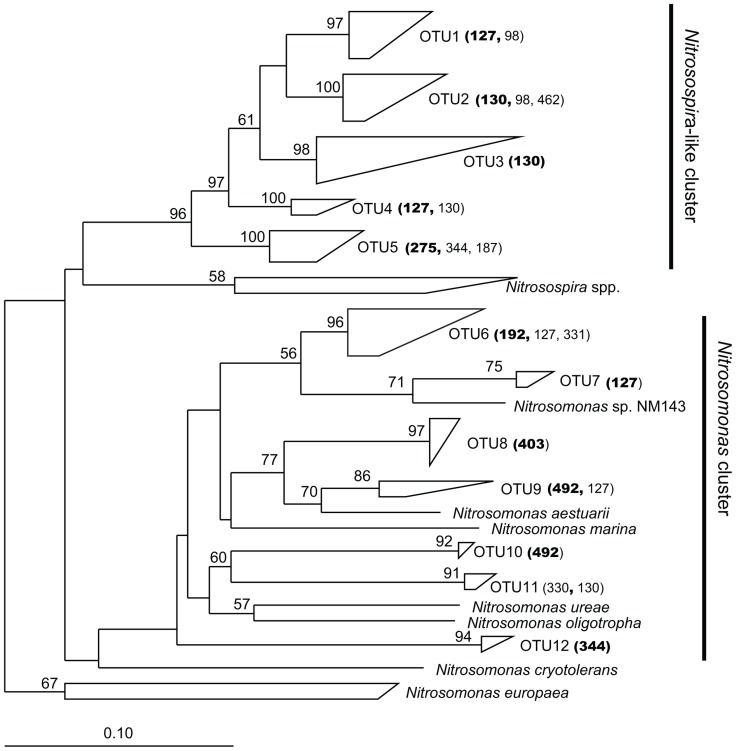
**Phylogenetic relationships among betaproteobacterial *amo*A nucleotide sequences recovered from plots in the Great Sippewissett Marsh**. The tree was inferred from 466 nucleotides using the neighbor-joining algorithm with the Kimura two-parameter correction. Bootstrap values >50% are shown at the internal nodes. Each polygon represents sequences from a single OTU with the TRF sizes of the sequences (number of base pairs determined *in silico*) indicated parenthetically. TRF sizes representing ≥85% of the sequences in the cluster are shown in bold.

Coverage of the betaproteobacterial *amo*A gene clone libraries ranged from 89.8 to 100% and we detected a total of 12 OTUs that closely matched the TRFs (**Figure 3**). Sixty-one percent of the sequences were affiliated with the uncultured *Nitrosospira*-like cluster, with the remainder affiliated with the *Nitrosomonas* cluster.

### RESPONSES OF AOB POPULATIONS TO FERTILIZATION

We evaluated relative abundance of each TRF in relation to fertilization rates to characterize responses of specific AOB populations. Four TRFs were significantly correlated with fertilization rates (**Table 1**), with TRF492 showing the strongest positive response to fertilization. Distribution of sequences among the fertilized treatments corroborated the TRF patterns. For example, all 31 sequences representing TRF 492 in OTUs 9 and 10 were recovered exclusively from HF and XF plots.

**Table 1:** Pearson’s correlation coefficients (*r*) describing the relationships between abundance of TRFs and fertilization rates.

TRF size	Correlation with fertilization rates	Lineage
**AOB**
TRF130	(0.50)	*Nitrosospira*-like
TRF403	0.55	*Nitrosomonas aestuarii/marina*
TRF472	0.42	*Nitrosomonas eutropha*
TRF492	0.76	*Nitrosomonas ureae/oligotropha*
**AOA**
TRF160	0.48	*Nitrosopumilus* group 1

*Only significant correlations (*P* ≤ 0.05) are shown. Negative correlations are indicated parenthetically.*

**FIGURE 4:**
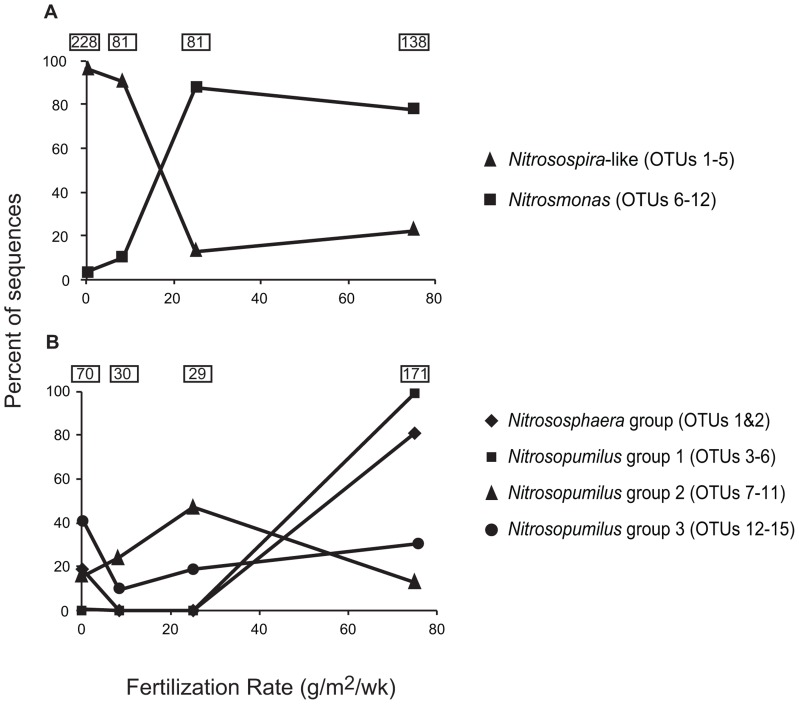
**Distribution of betaproteobacterial *amo*A clones **(A)** and archaeal *amo*A clones **(B)** related to fertilization rates**. Groups are based on phylogenetic clusters designated in **Figures 3 and 5**. The number of sequences used to generate data points for each fertilization rate are shown in boxes.

We also observed a striking shift in the distribution of *Nitrosospira*-like and *Nitrosomonas*-related sequences related to fertilization rates (**Figure 4A**). Ninety-one percent of the *Nitrosospira*-like sequences were recovered from C and LF sites, while *Nitrosomonas* sequences were found mostly in HF and XF sites.

### SHIFTS IN AOA COMMUNITY COMPOSITION

**FIGURE 5:**
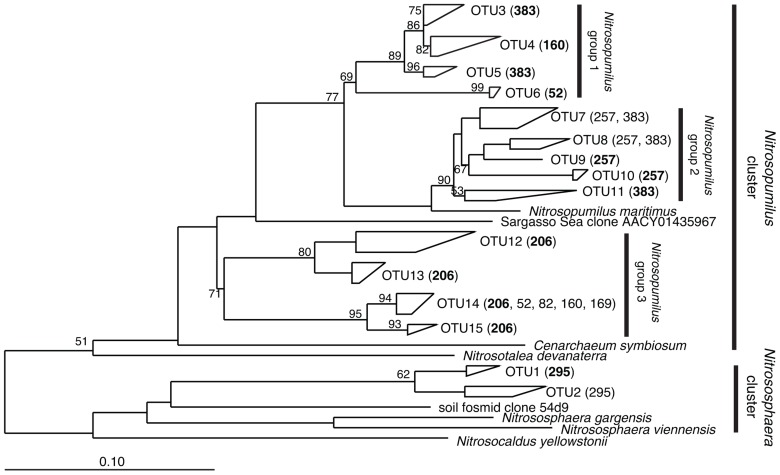
**Phylogenetic relationships among archaeal *amo*A nucleotide sequences recovered from plots in the Great Sippewissett Marsh.** The tree was inferred from 569 nucleotides using the neighbor-joining algorithm with the Kimura two-parameter correction. Bootstrap values >50% are shown at the internal nodes. Each polygon represents sequences from a single OTU with the TRF sizes of the sequences (number of base pairs determined *in silico*) indicated parenthetically. TRF sizes representing ≥85% of the sequences in the cluster are shown in bold.

A total of 12 AOA TRFs were identified among all plots. TRF 383 was the most abundant overall in both the low and high marsh habitats (23.9 and 39.9% relative abundance, respectively) and 29.8% of the sequences recovered from all the sites had this predicted TRF. This is also one of two TRFs (along with TRF 257) representing sequences most closely related to *Nitrosopumilus maritimus* (**Figure 5**). TRF52 was identified from sequence data (**Figure 5**), but was not included in the TRFLP profile because we could not discriminate actual signal from background for TRFs smaller than 70 bp.

Non-metric multidimensional scaling ordination of archaeal communities based on TRFLP data suggest some effect of fertilization treatment, but the patterns were not robust (**Figure 2B**). There were distinct AOA communities in the control and XF plots in the high marsh sites, but the patterns among the low marsh samples were less convincing. Furthermore, unlike the results for AOB communities, we found no significant correlations of ordination scores for axis 1 (or 2) with fertilization rates, N loading rates, or salinity.

**FIGURE 6:**
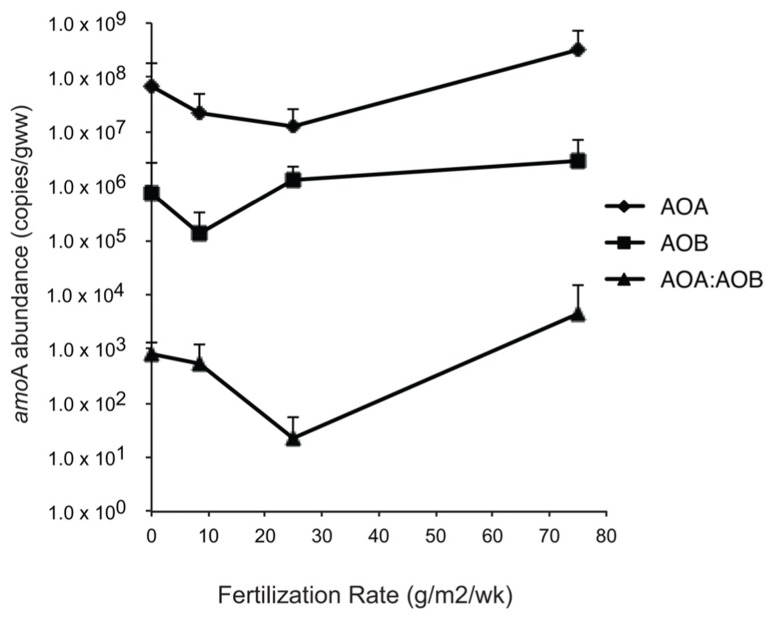
** Abundance of betaproteobacterial and archaeal *amo*A genes and ratios of *amo*A genes relative to levels of fertilization**.

Analysis of archaeal *amo*A gene sequences revealed 15 OTUs (**Figure 5**). Although a few of the phylogenetic clusters were characterized by a unique TRF, there was generally less agreement between AOA TRF and OTU distribution compared to AOB. The majority of sequences were affiliated with the water column/sediment cluster as designated by Francis et al. (2005), more recently defined as the *Nitrosopumilus* cluster described by Pester et al. (2012); **Figure 6**). Twenty-one sequences fell within the *Nitrososphaera* cluster as identified by Pester et al. (2012), a subset of the soil/sediment cluster. Coverage of archaeal *amo*A gene diversity in the clone libraries ranged from 92.9 to 100%.

### RESPONSE OF AOA POPULATIONS TO FERTILIZATION

Contrary to patterns observed for AOB, only one TRF showed a significant correlation with fertilization rates (**Table 1**). However, there were more distinct patterns within the sequence data (**Figure 4B**). We identified a subcluster of 109 sequences within the *Nitrosopumilus* cluster that was composed exclusively (with the exception of a single sequence) of sequences recovered from XF plots. We also observed a similar pattern for sequences affiliated with the *Nitrososphaera* cluster.

### AOA AND AOB ABUNDANCE

Abundance of betaproteobacterial *amo*A genes ranged from 4.5 × 10^3^ to 1.3 × 10^7^ gene copies/g sediment, and was one to three orders of magnitude lower than archaeal *amo*A abundance (**Figure 6**). Although abundance was highest in the XF plots, the differences among mean values were not significant and linear regression analysis also confirmed no significant fertilization effect.

Abundance of archaeal *amo*A genes ranged from 2.0 × 10^5^ to 1.2 × 10^9^ gene copies/g sediment (wet weight) and were not significantly different among fertilization treatments. Linear regression analysis also confirmed no significant fertilization effect. Ratios of AOA:AOB abundance ranged from 0.5 to 3.1 × 10^4^ and were highest at the XF plots (**Figure 6**), although the differences were not significant.

To account for possible differences in DNA extraction efficiencies, we normalized AOB and AOA *amo*A gene abundances to numbers of bacterial 16S rRNA gene copies and to μg DNA (**Table 2**). Patterns of abundance for normalized data were virtually identical to patterns based on copy numbers per gram of sediment (data not shown).

**Table 2:** Mean (±SE) betaproteobacterial (AOB) and archaeal *amo*A (AOA) gene abundances presented as gene copies per μg DNA or normalized to copies of bacterial 16S rRNA genes in plots receiving different levels of fertilization.

Fertilization	AOA/μg DNA	AOB/μg DNA	AOA: bacterial 16S rRNA	AOB: bacterial 16S rRNA
Control	5.8 ± 2.8 × 10^6^	5.9 ± 5.3 × 10^4^	4.1 ± 2.1 × 10^-^^3^	2.3 ± 1.8 × 10^-^^5^
LF	2.0 ± 1.1 × 10^6^	1.1 × 10^4^ ± 6.8 × 10^3^	9.2 ± 3.9 × 10^-^^4^	5.1 ± 2.2 × 10^-^^6^
HF	1.3 × 10^6^ ± 6.0 × 10^5^	2.1 ± 1.5 × 10^5^	4.1 ± 1.9 × 10^-^^4^	4.7 ± 1.8 × 10^-^^5^
XF	3.5 ± 1.5 × 10^7^	3.2 ± 1.7 × 10^5^	4.12 ± 1.4 × 10^-^^2^	2.1 ± 1.1 × 10^-^^4^

## DISCUSSION

The effects of long-term fertilization in the Great Sippewissett Marsh have been documented for many of the major biological components of the marsh over the past four decades, elucidating fundamental mechanisms driving salt marsh ecology and environmental change. However, studies investigating the effects of fertilization on microbial communities have been lacking. Understanding how microorganisms respond to fertilization is important not only for predicting how microbially mediated ecosystem services may be altered by anthropogenically driven changes to the environment, but also for increasing our understanding of fundamental ecological principles that drive community dynamics. Just recently, Bowen et al. (2011) published the first study on microbial responses in the same plots that were sampled in our study and, rather surprisingly, found no fertilization effect on the composition of total bacterial or denitrifying communities. However, it is possible that there are differential responses among some microbial groups that were not detected in the analysis by Bowen et al. (2011), warranting additional studies within the marsh microbial community, particularly given the significant differences reported for some nitrogen-cycling processes in the marsh (Van Raalte et al., 1974; Hamersley and Howes, 2005). Our investigation of ammonia-oxidizing microorganisms in the marsh suggest, in fact, a complex pattern of responses that provide further insights into mechanisms controlling nitrifying populations and communities in coastal ecosystems.

### AOB COMMUNITY COMPOSITION AND RESPONSE TO FERTILIZATION

We found significant shifts in AOB communities that corresponded to fertilization, which is in agreement with several previous studies of AOB in fertilized soils and sediments (Chu et al., 2007; Shen et al., 2011; Wu et al., 2011). Our results, however, are somewhat contrary to the recent study by Lage et al. (2010) who found shifts in salt marsh AOB community composition only when a single nutrient (N or P) was applied, but not when both were applied together. Since the fertilizer applied in our study is a complex mix, it is difficult to attribute changes in community composition to a particular nutrient. However, our fertilization rates were 4–12 times higher than those used by Lage et al. (2010), and may account for the differences in community response.

The distribution of sequences we recovered for the betaproteobacterial *amo*A gene was generally similar to distributions found from other estuarine and salt marsh studies, with the majority of sequences falling into the *Nitrosospira*-like cluster (see Bernhard and Bollmann, 2010 for review). Sequences within this cluster were recovered from all fertilization treatments as well as both marsh habitats, suggesting that AOB within this cluster are either physiologically diverse or have high physiological plasticity.

Conversely, all of the OTUs within the *Nitrosomonas* group were restricted by marsh habitat or fertilization treatment, with the exception of OTU 7, suggesting that *Nitrosomonas*-related AOB in the marsh are more specialized compared to their *Nitrosospira* counterparts. Particularly striking was the lack of sequences from control or LF sites among OTUs affiliated with the *Nitrosomonas aestuarii/marina* and the *Nitrosomonas ureae/oligotropha* clusters. Other studies of AOB in fertilized or polluted sediments have also found AOB communities to be dominated by *Nitrosomonas*-related sequences (Beman and Francis, 2006; Cao et al., 2011a,b).

Identification of specific AOB populations that showed consistent response to fertilization may help to better characterize the ecophysiology of uncultured AOB, and may serve as important clues to assist in the ultimate cultivation of these culturally recalcitrant AOB. The differential responses of specific AOB populations also suggest mechanisms for niche-differentiation within this relatively constrained phylogenetic group. Such distinct and reproducible patterns of AOB community composition in relation to fertilization also support the use of AOB as bioindicators, as has been previously suggested (Dang et al., 2010).

### AOA COMMUNITY COMPOSITION AND RESPONSE TO FERTILIZATION

The lack of a consistent response of the AOA community composition to fertilization was somewhat unexpected given the fertilization history and significant differences in nitrogen-processing rates previously reported. However, results from other studies of AOA community composition in response to fertilization report a similar lack of response. Wu et al. (2011) found that long-term fertilization (22 years) with urea did not alter AOA communities in a paddy soil, and Verhamme et al. (2011) found that AOA communities in soils were not different from controls after short-term (28 days) ammonium additions. We detected distinct AOA communities related to fertilization in the high marsh, but because the salinity in the XF high marsh plots was significantly lower than in the control plots, the shift in AOA communities in the high marsh may be due to differences in salinity rather than fertilization. Salinity has been shown previously to correlate with changes in AOA abundance (Moin et al., 2009) and community composition (Sahan and Muyzer, 2008). It is also possible that the AOA community may respond to fertilization at other times of the year as their requirements change with seasonal environmental changes. Seasonal shifts in AOA communities have been reported in some estuaries (Sahan and Muyzer, 2008).

Although we did not detect robust patterns among the AOA communities related to fertilization rates based on TRFLP data, we identified some patterns among the sequence data suggesting a possible fertilization effect. OTUs 3, 4, 5, and 6 (*Nitrosopumilus* group 1 in **Figure 5**) were composed exclusively (with one exception) of sequences recovered from XF sites, suggesting that these AOA may be well-adapted to high nutrient conditions. Unfortunately, some of the TRFs representing OTUs within *Nitrosopumilus* group 1 were also found in other OTUs, thus obscuring any patterns related to fertilization in the TRFLP analysis. Sequences representing the *Nitrososphaera* group were also dominant among XF plots, but the number of sequences in this group was relatively small compared to other groups. Additionally, the sequences we recovered from both *Nitrosopumilus* group 1 and the *Nitrososphaera* group were closely related to AOA recovered from environments that were not nutrient-enriched, indicating that the sequences in these clusters are not necessarily indicative of high nutrient conditions.

Environmental factors identified as potentially important in regulating AOA community composition are many and include salinity, temperature, pH, nitrite, dissolved oxygen, net primary productivity, and some heavy metals (reviewed in Erguder et al., 2009). However, common factors regulating AOA communities in coastal systems have not clearly emerged, suggesting that AOA may be a more physiologically diverse group relative to the AOB and may respond differentially to changing environmental conditions. Other studies in marine environments suggest that AOB and AOA respond to different environmental cues (Hollibaugh et al., 2011; Bouskill et al., 2012), but exactly what the cues for AOA are in salt marshes remain unclear.

### AOA AND AOB ABUNDANCE

Abundances of AOA and AOB were within the ranges of abundances reported in other estuarine and salt marsh studies (Bernhard and Bollmann, 2010). However, we were somewhat surprised by the lack of robust and consistent fertilization effects on either AOA or AOB abundance, particularly given what we know about increases in nitrogen-processing rates in the fertilized plots. Although we did detect a significant fertilization effect for AOA, the pattern of abundance was not compelling, suggesting something other than fertilization is regulating abundance. Others have reported significant changes in AOA and AOB abundance in response to fertilization in agricultural soil systems (Chu et al., 2008; Kelly et al., 2011; Shen et al., 2011; Wu et al., 2011), but Lage et al. (2010) reported no effect of fertilization on AOB abundance in a salt marsh. The lack of a compelling change in abundance in our fertilized plots is that AOA and AOB may be limited by some other factor, such as pH, oxygen, or sulfide. Sulfide levels can be quite high in salt marsh sediments due to tidal flooding, and effects of sulfide on nitrification are well-documented (Joye and Hollibaugh, 1995; Caffrey et al., 2010). Periods of anoxia may also be quite frequent and may further limit nitrification and thus, abundance of nitrifiers.

The lack of a consistent and robust response of AOA and AOB abundance to differences in fertilization may also be due to the complex nature of the fertilizer applied. Differential responses of AOA and AOB populations to different components of the fertilizer may diminish any effects on overall abundance. Studies that isolate individual environmental factors may be necessary to determine the effects of specific components on the AOA and AOB.

## CONCLUSION

Our results revealed differential responses of AOA and AOB communities and abundance to long-term fertilization in a New England salt marsh and provide some insight into population dynamics, particularly among AOB, and potential regulatory factors of nitrifiers in the marsh. AOB communities showed robust and consistent responses to fertilization, while responses of AOA communities were less clear, possibly reflecting differences in physiological tolerances. However, abundance patterns of AOA and AOB showed minimal responses to fertilization treatments, suggesting that factors other than nutrients, such as redox conditions, may limit abundance of these organisms in the marsh.

We find it notable that in spite of what we know about nitrogen cycling in the marsh, there was no compelling response of AOA or AOB abundance or AOA community composition to long-term fertilization. This leads us to suspect that either the methods are not inclusive of all nitrifiers in the marsh or that some targeted populations are not obligate nitrifiers. Additionally, since the metabolic pathway for ammonia oxidation in archaea has not been fully elucidated, it is possible that the archaeal *amo*A gene is not an appropriate marker for characterizing AOA populations. However, given the equally surprising results reported by Bowen et al. (2011) showing no response of denitrifying communities using different methods to analyze samples from the same plots used in our study, we are forced to consider that we are missing a critical link between community composition and abundance and nitrogen processes in the marsh.

## Conflict of Interest Statement

The authors declare that the research was conducted in the absence of any commercial or financial relationships that could be construed as a potential conflict of interest.
